# Cerebellar and Cerebral Amyloid Visualized by [^18^F]flutemetamol PET in Long-Term Hereditary V30M (*p.V50M*) Transthyretin Amyloidosis Survivors

**DOI:** 10.3389/fneur.2022.816636

**Published:** 2022-03-04

**Authors:** Erica Irene Uneus, Christer Wilhelmsson, David Bäckström, Intissar Anan, Jonas Wixner, Björn Pilebro, Katrine Riklund, Mattias Ögren, Margareta Ögreen, Jan Axelsson, Ole B. Suhr, Torbjörn Sundström

**Affiliations:** ^1^Section of Neurology, Department of Clinical Science, Neurosciences, Umeå University, Umeå, Sweden; ^2^Department of Community Medicine and Rehabilitation, Geriatric Medicine, Umeå University, Umeå, Sweden; ^3^Department of Public Health and Clinical Medicine, Umeå University, Umeå, Sweden; ^4^Wallenberg Centre for Molecular Medicine, Umeå University, Umeå, Sweden; ^5^Department of Public Health and Clinical Medicine, Heart Centre, Cardiology, Umeå University, Umeå, Sweden; ^6^Department of Radiation Sciences, Diagnostic Radiology, Umeå University, Umeå, Sweden; ^7^Department of Radiation Sciences, Radiation Physics, Umeå University, Umeå, Sweden

**Keywords:** amyloidosis-hereditary, amyloid angiopathy, [^18^F]flutemetamol, positron emission tomography, transthyretin

## Abstract

**Introduction:**

Hereditary transthyretin (ATTRv) amyloidosis caused by the V30M (*p. V50M*) mutation is a fatal, neuropathic systemic amyloidosis. Liver transplantation has prolonged the survival of patients and central nervous system (CNS) complications, attributed to amyloid angiopathy caused by CNS synthesis of variant transthyretin, have emerged. The study aimed to ascertain amyloid deposition within the brain in long-term ATTRv amyloidosis survivors with neurological symptoms from the CNS.

**Methods:**

A total of 20 patients with ATTR V30M having symptoms from the CNS and a median disease duration of 16 years (8–25 years) were included in this study. The cognitive and peripheral nervous functions were determined for 18 patients cross-sectionally at the time of the investigation. Amyloid brain deposits were examined by [^18^F]flutemetamol PET/CT. Five patients with Alzheimer's disease (AD) served as positive controls.

**Result:**

60% of the patients with ATTRv had a pathological Z-score in the cerebellum, compared to only 20% in the patients with AD. 75% of the patients with transient focal neurological episodes (TFNEs) displayed a pathological uptake only in the cerebellum. Increased cerebellar uptake was related to an early age of onset of the ATTRv disease. 55% of the patients with ATTRv had a pathological Z-score in the global cerebral region compared to 100% of the patients with AD.

**Conclusion:**

Amyloid deposition within the brain after long-standing ATTRv amyloidosis is common, especially in the cerebellum. A cerebellar amyloid uptake profile seems to be related to TFNE symptoms.

## Introduction

The aggregation, distribution, and neurotoxicity of deposited amyloid species in the brain are well-described for the most common neurodegenerative disorder, Alzheimer's disease (AD) (1), but are much less studied in hereditary transthyretin amyloid (ATTRv) amyloidosis. ATTRv is a fatal systemic amyloidosis caused by mutations in the transthyretin (TTR) gene. Amyloid deposition and symptoms are noted for numerous organs such as the peripheral nervous system, heart, eyes, kidneys, and gastrointestinal tract, but rarely for the central nervous system (CNS) (2–4). Currently, more than 140 various mutations are reported with wide variation in the phenotype between the different mutations and within the same mutation (5).

The mechanism behind the phenotypical variation between various mutations has not been established. However, it has been suggested that the combination of low serum levels of mutant relative to wild type TTR together with a mutation that produces a low kinetic and thermodynamic stability of the TTR tetramer predispose to CNS complication. This has been shown for the D18G (*pD38G)* and A25T (*p.A45T*) TTR mutations (6, 7). Since this is not the case for the V30M (*p.V50M*) mutation, this may explain the low occurrence of CNS complications in the natural history of patients with ATTR V30M.

V30M (*p.V50M*) gives rise to a predominantly neuropathic disease but the phenotype varies. The neuropathic phenotype with early onset (below the age of 50) is associated with an otherwise rarely found type of transthyretin amyloid (ATTR) fibril that consists only of full-length TTR (type B). However, the phenotype characterized by later onset and neuropathy, and cardiomyopathy, is associated with the more common fibril type of a mixture of fragmented and full-length ATTR fibrils (type A) (8).

The disease is relentlessly progressive, and before 1990 when liver transplantation (LTx) was introduced as a treatment, the median survival was 10–13 years for Swedish patients (2, 9). The rationale for LTx was to replace the amyloidogenic mutant TTR produced by the liver with a liver that produced wild-type TTR only. The outcome was encouraging, but continuous deterioration, especially of heart function, was noted for patients with non-TTR V30M and late-onset V30M; this was probably related to amyloid fibril composition (10–12).

Later on, medical treatments have become available, i.e., TTR tetrameric stabilizers since 2011, and recently gene silencing (13–16). However, none of the current treatments affect the variant TTR synthesized by the choroid plexus of the brain or by the retina of the eye, and continuous amyloid formation in the eye has been reported for LTx and medically treated patients (17–19) and also continuous amyloid formation in the leptomeninges after LTx in the TTR Y114C (*p.134Y*) mutation, a mutation characterized by CNS complications in its natural history (17). In addition, amyloid deposition derived from variant TTR within the brain of patients with long-term surviving LTx was reported from two studies where CNS symptoms attributed to cerebral amyloid angiopathy (CAA) were commonly encountered (20, 21).

The improved survival may provide the necessary time needed for amyloid formation from variant TTR within the brain. However, atrial fibrillation and embolization leading to transient ischemic attack (TIA) and stroke is also common complication in patients with ATTR V30M, and it may be difficult to separate from symptoms of amyloid angiopathy (22).

Detection of ATTR-type CAA by MR examination with gadolinium enhancement has been used to identify meningeal ATTR (23). Further, PET [^11^C]Pittsburgh component P ([^11^C]PIB) examination has been used successfully to assess beta-amyloid deposition in the brains of patients with AD and patients with ATTRv (21, 24, 25) and to detect cardiac ATTR (26, 27). However, [^11^C]PIB's short half-life has promoted the development of newer tracers with longer half-lives and equal imaging properties (28).

Magnetic resonance imaging is suggested to be the most reliable tool in identifying CAA according to modified Boston criteria (29). Since even MRI-secure pacemakers complicate MRI examination, PET with an amyloid tracer appears to be an attractive alternative to detect brain amyloid depositions in patients with ATTRv.

The objective of this study was to visualize the amyloid deposition within the brain in patients with long-standing ATTR V30M amyloidosis and symptoms from the CNS, using PET/CT examinations with a more stable tracer ([^18^F]flutemetamol). Our primary aim was to evaluate any correlations between the brain amyloid deposition and the symptoms and other disease characteristics of patients. A secondary aim was to see if the amyloid deposition in patients with ATTRv amyloidosis could be distinguished from that of patients with AD. Therefore, patients with AD served as positive controls.

## Patients and Methods

### Patients

In this cross-sectional observational study, 27 long-term V30M ATTR amyloidosis survivors (≥ 8 years after diagnosis) in the counties of Västerbotten and Norrbotten, Sweden, with symptoms from the CNS were identified. Their CNS symptoms should have had an onset after the diagnosis. All the patients have had their diagnosis settled by genetic testing and a positive biopsy for ATTR. In 11 patients, the fibril type had been determined by Western blot analysis (8).

Seven patients did not participate in this study for various reasons that included dementia and advanced disease. Thus, 20 patients underwent PET/CT examination and their medical records were reviewed. Eighteen patients were liver transplanted and the immune suppression regimes were based on tacrolimus for 13 patients, and on cyclosporine A for 4 patients. Data for one patient were not available. Two patients had been treated by TTR stabilizers (diflunisal or tafamidis). No patient had diabetes and no patient had kidney failure requiring dialysis [four patients had an estimated glomerular filtration rate (eGFR), below 60 ml/min/1.73 m^2^ based on the CKD-EPI equation]. Clinical data are given in Table 1.

**Table 1:** Clinical data of the 20 patients with hereditary transthyretin amyloid amyloidosis.

Males/females	5/15
Age at examination, yrs (median and range)	65 (42–79)
Age at disease onset, yrs (median and range)	47 (28–64)
Late/early onset^a^	9/11
Amyloid fibril type: A/B^b^	0/11
**Duration of disease, yrs (median and range)**	
From onset From diagnosis	17.5 (10–27) 16.5 (9–26)
Treatment, transthyretin-stabilizer/liver transplantation	2/18
Duration from liver transplantation, yrs (median and range)	14.5 (8–23)
Duration of transthyretin stabilizer treatment, yrs	12 and 17
**PND^c^-score**	
I II IIIA	8 9 3
Atrial fibrillation/anticoagulation therapy	7/7
Pacemaker treatment	8
Kidney function^d^; eGFR^e^ (median and range)	73.5 (35–90)
**Eye-complications**	
Glaucoma Vitreous opacities and glaucoma	3 5

a *Early onset is defined as the onset of symptoms before the age of 50*.

b *A denotes fibrils consisting of a mixture of fragmented and full-length transthyretin; B denotes fibrils consisting of only full-length transthyretin. Nine patients had not had their amyloid fibril composition determined*.

c *Peripheral Neuropathy Disability score, where I denotes sensory disturbances in the lower limbs, but preserved walking capability, II denotes impaired walking capability, but the ability to walk without support, and IIIA denotes walking with the aid of one stick or crutch*.

d *Data are not available for 3 patients*.

e *Estimated glomerular filtration rate (ml/min/1.73 m^2^)*.

One patient died from septicemia before the planned neurological assessment and one patient desired not to be informed of the outcome of the PET examination or to undergo neurological assessment. Thus, 18 patients underwent clinical evaluation.

Five male patients (age 58–75 years, 3–8 years of cognitive decline), all diagnosed with AD by ^18^F-fluorodeoxyglucose PET/CT examination and cerebrospinal fluid A-beta levels, were included as a positive control group.

### Methods

#### Clinical Evaluation

A total of 18 patients with ATTRv amyloidosis were evaluated with interviews and clinical examinations between October 2018 and April 2019 after completion of the PET/CT examination. The assessments were done by one examiner (co-author EU). The patients were interviewed for neurological symptoms, especially those indicating transient focal neurological episodes (TFNEs) (30). The peripheral neuropathy disability (PND) score (I–IV) was used to grade the neuropathy and mobilization of patients, where I denotes sensory disturbances in the lower limbs but preserved walking capability, and IV denotes confined to a wheelchair or bedridden (31). Cognitive assessment was made with the Montreal Cognitive Assessment (MoCA) score that has high sensitivity and specificity for mild cognitive impairment and mild AD (90–100% and 87–100, respectively) when a cut-off score of 26 is used (32). The neuropathy was assessed by the Neuropathy Impairment Score-Lower Limbs (NIS-LL), which is well-validated as a reliable measure of ATTR neuropathy (33).

This study did not include concomitant MR brain imaging. Eight patients wore a pacemaker, which prevents MR examination for research purposes. A majority of the patients (13/20) had undergone a CT or MR examination within 4 years. Six of these patients had MR imaging (of which three were with gadolinium contrast), and seven had CT brain imaging (of which four showed ischemic stroke). One of the three patients who had MR imaging with gadolinium contrast had a pronounced leptomeningeal enhancement.

#### PET/CT

All the PET/CT examinations were acquired with a GE Discovery 690 PET/CT scanner (General Electric, Wisconsin, USA). The participants were injected intravenously with 173–195 MBq (4.7–5.3 mCi) [^18^F]flutemetamol that was manufactured according to PET Current Good Manufacturing Practice (cGMP) standards (34, 35). Following a low-dose CT (120 kV, 10 mA, 0.8 s rotation time), a 30-min PET scan of the brain was performed that started 90 min postinjection. All the PET images were reconstructed to a voxel size of 1.95 × 1.95 × 3.27 mm^3^ with the VuePoint SharpIR iterative algorithm (6 iterations, 24 subsets, 3.0 mm cutoff filter) employing time-of-flight, decay, attenuation, and scatter correction (28, 36).

Data were analyzed with the CortexID software (GE Healthcare) that compares brain regions to a built-in atlas of healthy (age 30–85 years) ^18^F-flutemetamol scans (37). Relative uptake is quantified for each region as the uptake is divided by the uptake in the reference region pons. The Z-score relative to the healthy-subject database is calculated for each region. In addition, a global composite Z-score for multiple brain regions is calculated (38). A Z-score above two is classified as an abnormally increased amyloid burden. Cortex-ID has no age correction for [^18^F]flutemetamol because amyloid density measured by amyloid PET in negative patients has a very weak correlation with the age of the patient.

#### Statistical Methods

Non-parametrical methods were used for descriptive and statistical analysis. Differences between groups were analyzed by the Mann–Whitney *U* test and correlations by the Spearman's test. A *P*-value below 0.05 was accepted as statistically significant.

#### Ethics

This study was approved on April 4, 2017 by the regional Ethics Committee at the Umeå University (2017/84-31). All the patients, and when appropriate for patients with AD a close relative, were informed orally and in writing of this study, and all had agreed to participate and signed a consent form.

## Results

**Figure 1:**
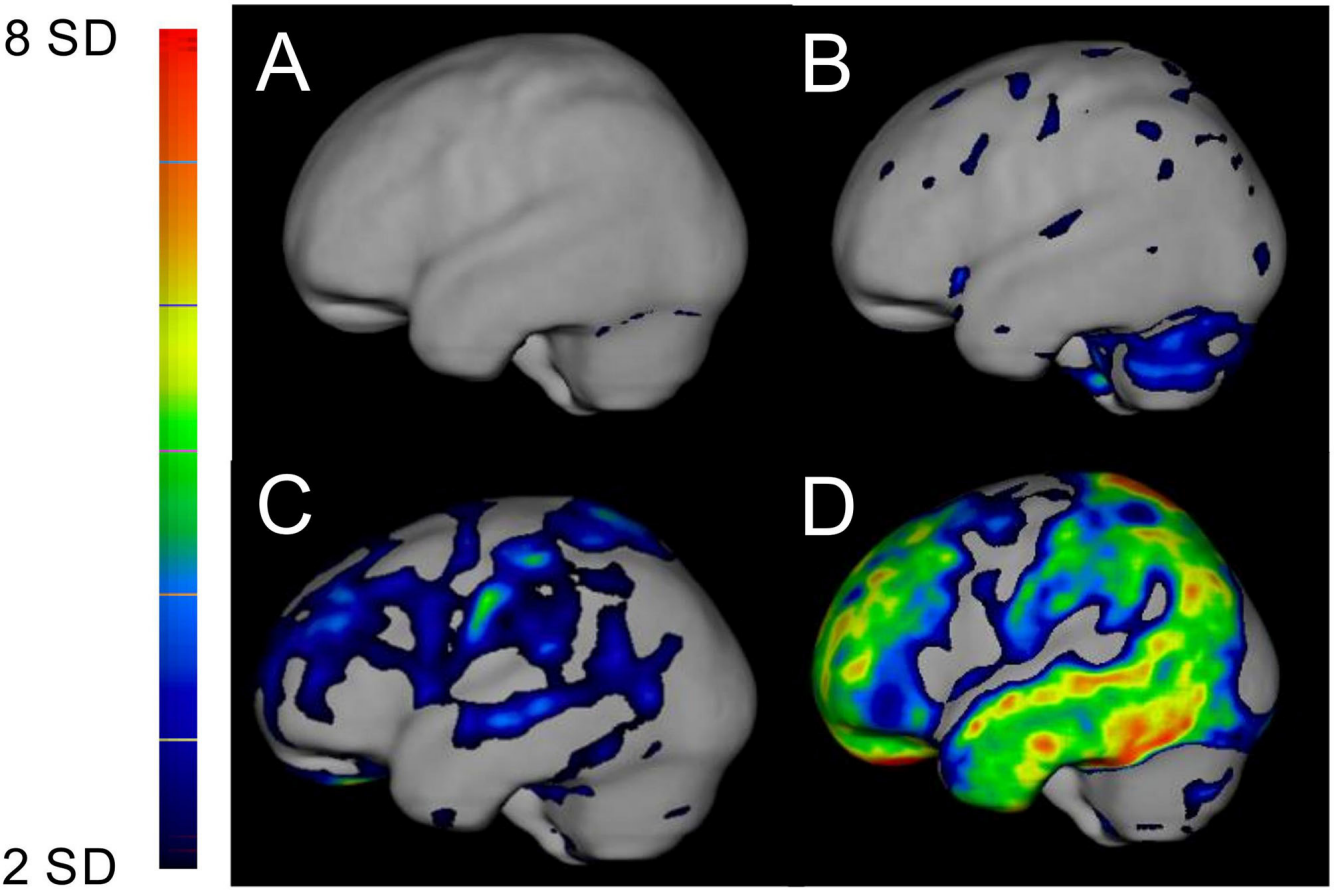
PET/CT examination of [^18^F]flutemetamol shown as Z-score in the brain in relation to pons from a left lateral view of the brain. The intensity of the uptakes is visualized on the left panel and is graded from low increased uptake in blue (Z-score +2SD) to high increased uptake in red (Z-score +8SD). **(A)** An ATTR V30M patient with 15 years of disease showing no increased uptake; **(B)** an ATTR V30M patient with 16 years of disease showing increased uptake in the cerebellum, **(C)** an ATTR V30M patient with 27 years of disease showing diffuse increased uptake in the brain, and **(D)** an Alzheimer's patient with generalized increased uptake in the frontoparietotemporal regions.

The neurological symptoms and findings are shown in Table 2. All 11 the patients, who had had their ATTR fibril type determined had type B fibrils, and their [^18^F]flutemetamol uptake was not different from that of the remaining 9 patients; therefore, we believe that we have a homogeneous group of mostly type B patients. The outcome of [^18^F]flutemetamol PET is shown in Table 3 and given in Figure 1.

**Table 2:** Neurological symptoms and findings.

Headache/migraine	11
Stroke	4
Seizures/epilepsy	2
Memory impairment	8
Transient focal neurological episodes (TFNE)	4
NIS-LL^a^, points (median and range)	23 (2–42)
MoCA^b^, points (median and range)	26 (21–29)

a *Neurological impairment score in the lower limbs with a range from 0 to 88; where a higher score indicates higher impairment*.

b *Montreal cognitive assessment scale with a range from 0 to 30; where a lower score indicates higher cognitive impairment*.

**Table 3:** Outcome of [^18^F]flutemetamol PET/CT examination.

	**ATTR^**a**^ V30M amyloidosis**	**Alzheimer's**
Composite Z score, reference pons median (range)	2.2 (−0.3–5.0)	6.1 (5.0–9.0)^b^
Males/females, median (range)	2.9 (2.6–4.9) / 1.9 (−0.3–4.8)^c^	
**Correlation (r**_**S**_ **) between composite Z score and**		
MoCA-score^d^	0.08 (NS^e^)	
Age at onset	0.08 (NS)	
Age at examination	0.00 (NS)	
Duration of disease from		
Onset	−0.20 (NS)	
Diagnosis	−0.34 (NS)	
Liver transplantation	−0.31 (NS)	
Cerebellum Z-score reference pons median (range)	2.5 (-1,2–5.5)	0.4 (−0.2–1.1)^f^
Males/females, median (range)	3.5 (0.2–5.2)/2.4 (−1.2–4.45) NS	
**Correlation (r**_**S**_ **) between Z-score cerebellum and**		
Age at onset	−0.46 (*P* < 0.05)	
Age at examination	−0.41 (NS)	
Duration of disease from		
Onset	0.12 (NS)	
Diagnosis	0.11 (NS)	
Liver transplantation	0.08 (NS)	

a *ATTR, Transthyretin amyloid*.

b *Statistically more cerebral amyloid deposits in Alzheimer's compared with ATTR V30M patients, P = 0.001*.

c *Statistically more cerebral amyloid deposits in male compared with female patients, P = 0.026*.

d *Montreal cognitive assessment*.

e *NS, Not statistically significant*.

f *Statistically more amyloid deposition in the cerebellum of ATTR V30M patients compared with that of patients with Alzheimer's disease. P = 0.025*.

### Global Cerebral Amyloid

55% of the patients with ATTRv amyloidosis had an abnormally increased cerebral amyloid burden (global composite Z-score > 2) compared to 100% in the patients with AD.

The composite Z-score was significantly lower in the patients with ATTRv amyloidosis compared to the patients with AD. There was no correlation between the patients with ATTRv amyloidosis global composite Z-score and ATTRv disease duration, onset, time of diagnosis, or MoCA score (Table 3). However, the male patient displayed a higher uptake than female patients. Seventy-five percent of the patients with ATTRv had a pathological Z-score in either the global composite cerebral or cerebellar region.

### Cerebellar Amyloid

**Figure 2:**
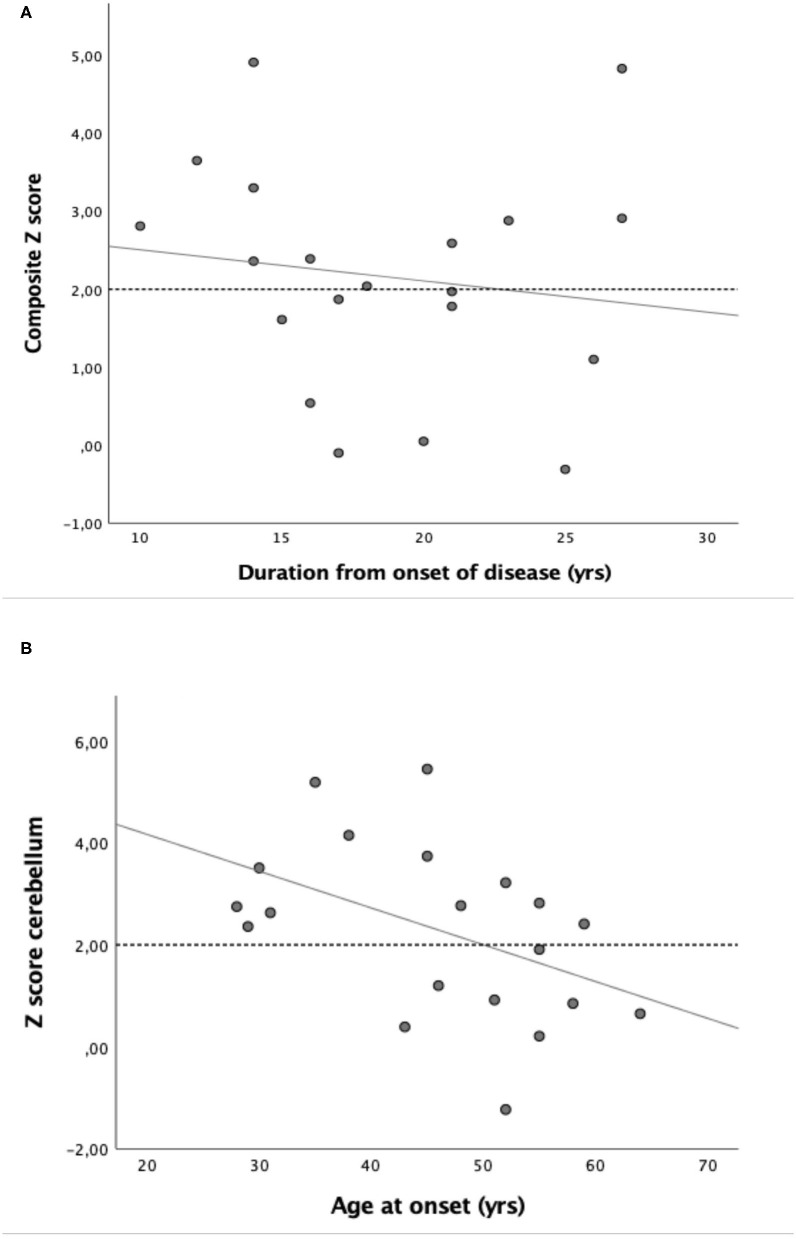
Scatter plot of the relationship between **(A)** composite Z-score and duration of disease from diagnosis (*r*_*S*_ = −0.20; NS) and **(B)** relationship between Z-score cerebellum and age at onset (*r*_*S*_ = −0.45; *P* < 0.05).

In the cerebellum, the Z-score was pathological (Z-score > 2) in 60% of the patients compared to 20% in the patients with AD. The patients with ATTRv amyloidosis had a significantly higher Z-score in the cerebellum than the patients with AD (*P* = 0.025). There was no correlation with the duration of the disease (Table 3). However, the cerebellar Z-score of patients with ATTRv amyloidosis was correlated with their age at disease onset, where younger patients showed a higher cerebellar [^18^F]flutemetamol uptake (Figure 2 and Table 3).

Figure 2 indicates that the patients with ATTR V30M appear to consist of two groups; one with an age of onset above 40 years in which the increased brain amyloid deposition (Z-score > 2) is evenly distributed between the different regions, and another group with a disease onset below the age of 40 in which the amyloid distribution is noted especially in the cerebellum. Five patients with ATTRv showed a pathological Z-score only in the cerebellum (and a non-pathological global composite Z-score). No significant difference in uptake between males and females was noted.

### Regional Difference in Amyloid Uptake

**Figure 3:**
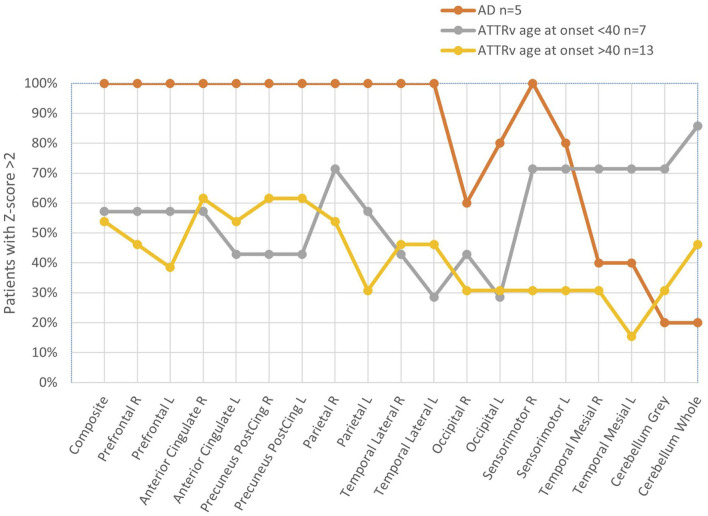
Fraction of patients with pathological Z-score (>2) in different brain regions. Curves represent patient groups: AD (orange), ATTR V 30M age <40 (gray), and ATTR V30M age >40 years (yellow).

In Figure 3, we have summarized the Z-score pattern for all the brain regions in patients with ATTRv below and over 40 years of age together with patients with AD. In contrast to the older group, the younger group shows a pattern with pathological Z-score in the cerebellum, sensorimotor cortex, and mesial temporal regions. In all the other regions, between 30 and 70% of the patients with ATTR V30M showed a pathological Z-score compared to almost 100% in the AD group. Interestingly, the younger age group (<40 years at onset) had a higher percentage of pathological Z-scores in the mesial temporal region compared to that of patients with AD.

No correlation was noted for any region between cognitive impairment (MoCA) and amyloid uptake (pre-frontal, anterior and posterior cingulate, precuneus, parietal, mesial and lateral temporal, occipital, sensorimotor, and cerebellum).

### Clinical Parameters and Outcome of PET

Four patients had transient focal neurological episodes (TFNEs) that included speech difficulties or aphasia, migrating paraesthesia in one arm, and for one patient a status epilepticus. Interestingly, three of the four patients with TFNEs belonged to the group of five patients with pathological cerebellar uptake, but normal global composite Z-scores (the 4th patient with TFNE had no signs of CNS amyloid). In addition, the patient with abnormal contrast enhancement on MR examination and symptoms consistent with TFNEs displayed a high cerebellar Z-score (4.15), but a normal global composite Z-score (1.87). None of the patients with TFNEs, including the patient with status epilepticus, had a composite Z-score >2.

Four other patients displayed a radiological confirmed ischemic stroke. All of these patients had atrial fibrillation and a composite median Z-score of 2.42 (−0.1 to 4.84).

## Discussion

The main findings in this study were that even though the amyloid deposition in the brain, visualized with the amyloid tracer [^18^F]flutemetamol in long-term ATTR V30M survivors was higher compared with the healthy-subject database (28), not all the patients developed abnormal amyloid deposits in the brain, irrespective of long disease duration. Furthermore, the depositions were especially confined to the cerebellum, which was related to age at disease onset, but not to disease duration. The latter is in contrast to the findings reported from Japan, where the uptake was correlated with disease duration (21). Since Japanese and Portuguese patients with ATTR V30M are generally younger at their disease onset, this may explain the higher reported incidence of CNS complications in their populations (20, 21).

Our findings indicate that a subgroup of patients is more susceptible to brain ATTR amyloid formation. It can be speculated, that an early onset of the disease implies that the ability of the chaperone system to degrade misfolded proteins is impaired at a young age. We have previously shown, that livers from patients with ATTR V30M have an impaired ER/protein folding pathway with differences in gene expression of chaperones [(39) #70]. The situation may be similar in the TTR producing choroid plexus of the brain, i.e., that an early onset of the disease is associated with an early and a more profound dysfunction of the chaperone system.

### Cerebellar Uptake

We observed that the probability of pathological cerebellar uptake was three times higher in patients with ATTRv than in patients with AD. It may be speculated that the molecular structure (i.e., the strain conformation) of deposited amyloid in ATTRv is different from amyloid-β fibrils deposited in AD in such a way that amyloid seeding will preferentially target different cell populations and structures (i.e., occurring with a cerebellar distribution rather than occurring more diffusely in the neocortex as in AD) (40). However, the molecular and functional differences between brain amyloid formation in ATTRv and AD are not well-understood. The higher uptake in the cerebellum of patients with ATTRv amyloidosis compared with that of patients with AD was previously reported (21).

For patients characterized by CNS symptoms in the natural history of the disease, a poor outcome has generally been observed after LTx (12). However, for the Y114C, the outcome has been acceptable even though an increased meningeal amyloid accumulation after transplantation was noted (17, 41).

### Differentiating AD and ATTRv

A secondary aim of this study was to see if patients with ATTRv having CNS amyloid could be distinguished from patients with AD using PET/CT examinations. Since patients with ATTRv had a more pronounced cerebellar distribution than patients with AD, we suggest that a pathological cerebellar [^18^F]flutemetamol uptake in patients with ATTRv points to ATTR deposition rather than AD.

### TFNE and CAA

Three patients with TFNEs displayed pathological cerebellar uptake with non-significant global cerebral uptake, a finding that supports the clinical relevance of increased cerebellar uptake as a marker of ATTR CAA in patients with TFNEs. Interestingly, vascular amyloid depositions in the cerebellum, is usually confined to cerebellar cortical and leptomeningeal vessels (42), and a robust association between a strictly superficial cerebellar microbleed pattern and CAA has been noted (43).

CAA symptoms were suggested to be caused by an “amyloid spell” leading to micro-bleedings (44). Interestingly, microbleedings were previously been reported in long-term ATTR amyloidosis survivors (20, 45). It should be mentioned that [^11^C]PIB PET did not show increased uptake in a Japanese patient with biopsy verified amyloid depositions within the brain, but MR examination showed late gadolinium enhancement of the meninges (46). It would be interesting to compare amyloid PET with gadolinium contrast MR in these patients.

### Stroke and Atrial Fibrillation

Atrial fibrillation and cardiac embolization are well-recognized complications for this group of patients (22), and four of our patients with ischemic stroke had atrial fibrillation and were on anticoagulation therapy. Their composite Z-scores varied from normal to clearly increased. However, our findings suggest that if a PET examination with an amyloid tracer displays no increased amyloid depositions within the cerebellum, a search for intermittent atrial fibrillation should be considered.

We still treat atrial fibrillation with anticoagulation (22), but we recommend novel oral anticoagulants (NOAC), even though the evidence is lacking for a decreased bleeding risk for NOAC treated patients with ATTRv (47).

### Amyloid Fibril Type

All patients that had their amyloid fibril type determined had type B, i.e., full-length TTR. In a previous heart study, a higher affinity of [^18^F]flutemetamol and [^11^C]PIB was noted for patients with type B fibrils (26, 48). However, the fibril type in ATTR CAA has not been determined in any patient, and we do not know if the fibril type found in fat pad biopsies also represents the fibril type within the brain.

### Memory

We noted that memory disturbances were reported by nearly 50% of the patients. Although there was no correlation between composite Z and MoCA scores, the frequently reported memory disturbances merit further investigation. All patients with increased Z-score, and who complained of memory disturbances were offered further evaluation according to the study protocol.

### Limitations

We did not have a reference group of healthy age-matched controls. We acknowledged that the ethical consequences of investigating healthy individuals could be problematic, since it is well-recognized that accumulation of amyloid within the brain occurs years before the individual displays any symptoms of AD, so we did not achieve ethical permission to investigate healthy controls. It would also have been interesting to investigate patients with mutations characterized by CNS complications in the natural history of the disease; however, no such patients were available.

In the evaluation of cerebral amyloid load, the analysis method was adapted from that used for patients with AD. One limitation could be that ATTRv is a systemic amyloid disease affecting all regions probably including the reference pons region. This might lead to false-negative results since the method relies on a ratio toward the reference region. Some of the patients with a high cerebellar uptake might have an accompanying increased uptake in adjacent pons leading to an underestimated Z-score.

In addition, the amyloid fibril composition within the brain has not been determined in any patient and considering the lower affinity of the tracer for type A fibrils, patients with type A ATTR depositions within the bran may have been missed.

## Conclusion

Amyloid deposition within the brain after long-standing ATTRv amyloidosis is common, especially in the cerebellum. A cerebellar amyloid uptake profile seems to be related to TFNE symptoms suggestive of a possible CNS phenotype.

## Data Availability Statement

The raw data supporting the conclusions of this article will be made available by the authors on reasonable request and in accordance with Swedish data security rules.

## Ethics Statement

The studies involving human participants were reviewed and approved by the Regional Ethics Committee at Umeå University, Umeå, Sweden. The patients/participants provided their written informed consent to participate in this study.

## Author Contributions

OS, EU, IA, JW, BP, and TS: initiation and planning of the study. EU, CW, IA, MÖgren, MÖgreen, JA, OS, and TS: investigation and data acquisition. JA and TS: methodology. OS and EU: project administration. OS and KR: resources. EU: writing original draft. EU, CW, DB, IA, JW, BP, KR, JA, OS, and TS: writing—review and editing. All authors contributed to the article and approved the submitted version.

## Funding

This study was funded by grants from the Swedish Heart and Lung Foundation grant no. 20160787 (OS), an ALF-grant from the Region of Västerbotten, Hjärnfonden (DB), and from the patients' organization FAMY and FAMY Norbotten and the AMYL foundation (JW and IA).

## Conflict of Interest

The authors declare that the research was conducted in the absence of any commercial or financial relationships that could be construed as a potential conflict of interest.

## Publisher's Note

All claims expressed in this article are solely those of the authors and do not necessarily represent those of their affiliated organizations, or those of the publisher, the editors and the reviewers. Any product that may be evaluated in this article, or claim that may be made by its manufacturer, is not guaranteed or endorsed by the publisher.

## References

[1] Serpell LC. Alzheimer's amyloid fibrils: structure and assembly. Biochim Biophys Acta. (2000) 1502:16–30. 10.1016/S0925-4439(00)00029-6 10899428

[2] Andersson R. Familial amyloidosis with polyneuropathy. A clinical study based on patients living in northern Sweden. Acta Med Scand Suppl. (1976) 590:1–64. 1064291

[3] Benson MD Kincaid JC. The molecular biology and clinical features of amyloid neuropathy. Muscle Nerve. (2007) 36:411–23. 10.1002/mus.20821 17554795

[4] Plante-Bordeneuve V Said G. Familial amyloid polyneuropathy. Lancet Neurol. (2011) 10:1086–97. 10.1016/S1474-4422(11)70246-0 22094129

[5] Rowczenio D Wechalekar A. Mutations in Hereditary Amyloidosis. Available online at: http://amyloidosismutations.com/ (accessed January 20, 2022).

[6] Hammarstrom P Sekijima Y White JT Wiseman RL Lim A Costello CE. D18G transthyretin is monomeric, aggregation prone, and not detectable in plasma and cerebrospinal fluid: a prescription for central nervous system amyloidosis? Biochemistry. (2003) 42:6656–63. 10.1021/bi027319b 12779320

[7] Sekijima Y Hammarstrom P Matsumura M Shimizu Y Iwata M Tokuda T. Energetic characteristics of the new transthyretin variant A25T may explain its atypical central nervous system pathology. Lab Invest. (2003) 83:409–17. 10.1097/01.LAB.0000059937.11023.1F 12649341

[8] Ihse E Ybo A Suhr O Lindqvist P Backman C Westermark P. Amyloid fibril composition is related to the phenotype of hereditary transthyretin V30M amyloidosis. J Pathol. (2008) 216:253–61. 10.1002/path.2411 18729067

[9] Suhr O Danielsson A Holmgren G Steen L. Malnutrition and gastrointestinal dysfunction as prognostic factors for survival in familial amyloidotic polyneuropathy. J Intern Med. (1994) 235:479–85. 10.1111/j.1365-2796.1994.tb01106.x 8182405

[10] Ericzon BG Wilczek HE Larsson M Wijayatunga P Stangou A Pena JR. Liver transplantation for hereditary transthyretin amyloidosis: after 20 years still the best therapeutic alternative? Transplantation. (2015) 99:1847–54. 10.1097/TP.0000000000000574 26308415

[11] Gustafsson S Ihse E Henein MY Westermark P Lindqvist P Suhr OB. Amyloid fibril composition as a predictor of development of cardiomyopathy after liver transplantation for hereditary transthyretin amyloidosis. Transplantation. (2012) 93:1017–23. 10.1097/TP.0b013e31824b3749 22395298

[12] Suhr OB Larsson M Ericzon BG Wilczek HE FAPWTR's investigators. Survival after transplantation in patients with mutations other than Val30Met: extracts from the FAP world transplant registry. Transplantation. (2016) 100:373–81. 10.1097/TP.0000000000001021 26656838 PMC4732012

[13] Adams D Gonzalez-Duarte A O'Riordan WD Yang CC Ueda M Kristen AV. Patisiran, an RNAi therapeutic, for hereditary transthyretin amyloidosis. N Engl J Med. (2018) 379:11–21. 10.1056/NEJMoa1716153 29972753

[14] Benson MD Waddington-Cruz M Berk JL Polydefkis M Dyck PJ Wang AK. Inotersen treatment for patients with hereditary transthyretin amyloidosis. N Engl J Med. (2018) 379:22–31. 10.1056/NEJMoa1716793 29972757 PMC12611561

[15] Berk JL Suhr OB Obici L Sekijima Y Zeldenrust SR Yamashita T. Repurposing diflunisal for familial amyloid polyneuropathy: a randomized clinical trial. JAMA. (2013) 310:2658–67. 10.1001/jama.2013.283815 24368466 PMC4139164

[16] Coelho T Maia LF Martins da Silva A Waddington Cruz M Plante-Bordeneuve V Lozeron P. Tafamidis for transthyretin familial amyloid polyneuropathy: a randomized, controlled trial. Neurology. (2012) 79:785–92. 10.1212/WNL.0b013e3182661eb1 22843282 PMC4098875

[17] Ando Y Terazaki H Nakamura M Ando E Haraoka K Yamashita T. A different amyloid formation mechanism: de novo oculoleptomeningeal amyloid deposits after liver transplantation. Transplantation. (2004) 77:345–9. 10.1097/01.TP.0000111516.60013.E6 14966406

[18] Casal I Monteiro S Beirao JM. Tafamidis in hereditary ATTR amyloidosis – our experience on monitoring the ocular manifestations. Amyloid. (2016) 23:262–3. 10.1080/13506129.2016.1236332 27748624

[19] Sandgren O Kjellgren D Suhr OB. Ocular manifestations in liver transplant recipients with familial amyloid polyneuropathy. Acta ophthalmologica. (2008) 86:520–4. 10.1111/j.1600-0420.2007.01098.x 18435819

[20] Maia LF Magalhaes R Freitas J Taipa R Pires MM Osorio H. CNS involvement in V30M transthyretin amyloidosis: clinical, neuropathological and biochemical findings. J Neurol Neurosurg Psychiatry. (2015) 86:159–67. 10.1136/jnnp-2014-308107 25091367

[21] Sekijima Y Yazaki M Oguchi K Ezawa N Yoshinaga T Yamada M. Cerebral amyloid angiopathy in posttransplant patients with hereditary ATTR amyloidosis. Neurology. (2016) 87:773–81. 10.1212/WNL.0000000000003001 27466465

[22] Wange N Anan I Ericzon BG Pennlert J Pilebro B Suhr OB. Atrial fibrillation and central nervous complications in liver transplanted hereditary transthyretin amyloidosis patients. Transplantation. (2018) 102:e59–66. 10.1097/TP.0000000000001975 29019809 PMC5802266

[23] Blevins G Macaulay R Harder S Fladeland D Yamashita T Yazaki M. Oculoleptomeningeal amyloidosis in a large kindred with a new transthyretin variant Tyr69His. Neurology. (2003) 60:1625–30. 10.1212/01.WNL.0000065901.18353.AB 12771253

[24] Hellstrom-Lindahl E Westermark P Antoni G Estrada S. *In vitro* binding of [H]PIB to human amyloid deposits of different types. Amyloid. (2014) 21:21–7. 10.3109/13506129.2013.860895 24286359

[25] Klunk WE Engler H Nordberg A Wang Y Blomqvist G Holt DP. Imaging brain amyloid in Alzheimer's disease with Pittsburgh Compound-B. Ann Neurol. (2004) 55:306–19. 10.1002/ana.20009 14991808

[26] Pilebro B Arvidsson S Lindqvist P Sundstrom T Westermark P Antoni G. Positron emission tomography (PET) utilizing Pittsburgh compound B (PIB) for detection of amyloid heart deposits in hereditary transthyretin amyloidosis (ATTR). J Nucl Cardiol. (2018) 25:240–8. 10.1007/s12350-016-0638-5 27645889

[27] Takasone K Katoh N Takahashi Y Abe R Ezawa N Yoshinaga T. Non-invasive detection and differentiation of cardiac amyloidosis using (99m)Tc-pyrophosphate scintigraphy and C-Pittsburgh compound B PET imaging. Amyloid. (2020) 27:266–74. 10.1080/13506129.2020.1798223 32722948

[28] Vandenberghe R Van Laere K Ivanoiu A Salmon E Bastin C Triau E. 18F-flutemetamol amyloid imaging in Alzheimer disease and mild cognitive impairment: a phase 2 trial. Ann Neurol. (2010) 68:319–29. 10.1002/ana.22068 20687209

[29] Charidimou A Martinez-Ramirez S Reijmer YD Oliveira-Filho J Lauer A Roongpiboonsopit D. Total magnetic resonance imaging burden of small vessel disease in cerebral amyloid angiopathy: an imaging-pathologic study of concept validation. JAMA Neurol. (2016) 73:994–1001. 10.1001/jamaneurol.2016.0832 27366898 PMC5283697

[30] Charidimou A Baron JC Werring DJ. Transient focal neurological episodes, cerebral amyloid angiopathy, and intracerebral hemorrhage risk: looking beyond TIAs. Int J Stroke. (2013) 8:105–8. 10.1111/ijs.12035 23336261

[31] Suhr OB Holmgren G Steen L Wikstrom L Norden G Friman S. Liver transplantation in familial amyloidotic polyneuropathy. Follow-up of the first 20 Swedish patients. Transplantation. (1995) 60:933–8. 10.1097/00007890-199511150-00009 7491696

[32] Nasreddine ZS Phillips NA Bedirian V Charbonneau S Whitehead V Collin I. The montreal cognitive assessment, MoCA: a brief screening tool for mild cognitive impairment. J Am Geriatr Soc. (2005) 53:695–9. 10.1111/j.1532-5415.2005.53221.x 15817019

[33] Adams D Coelho T Obici L Merlini G Mincheva Z Suanprasert N. Rapid progression of familial amyloidotic polyneuropathy: a multinational natural history study. Neurology. (2015) 85:675–82. 10.1212/WNL.0000000000001870 26208957 PMC4553033

[34] Koole M Lewis DM Buckley C Nelissen N Vandenbulcke M Brooks DJ. Whole-body biodistribution and radiation dosimetry of 18F-GE067: a radioligand for in vivo brain amyloid imaging. J Nucl Med. (2009) 50:818–22. 10.2967/jnumed.108.060756 19372469

[35] Nelissen N Van Laere K Thurfjell L Owenius R Vandenbulcke M Koole M. Phase 1 study of the Pittsburgh compound B derivative 18F-flutemetamol in healthy volunteers and patients with probable Alzheimer disease. J Nucl Med. (2009) 50:1251–9. 10.2967/jnumed.109.063305 19617318

[36] Bettinardi V Presotto L Rapisarda E Picchio M Gianolli L Gilardi MC. Physical performance of the new hybrid PETCT Discovery-690. Med Phys. (2011) 38:5394–411. 10.1118/1.3635220 21992359

[37] Lundqvist R Lilja J Thomas BA Lotjonen J Villemagne VL Rowe CC. Implementation and validation of an adaptive template registration method for 18F-flutemetamol imaging data. J Nucl Med. (2013) 54:1472–8. 10.2967/jnumed.112.115006 23740104

[38] Thurfjell L Lilja J Lundqvist R Buckley C Smith A Vandenberghe R. Automated quantification of 18F-flutemetamol PET activity for categorizing scans as negative or positive for brain amyloid: concordance with visual image reads. J Nucl Med. (2014) 55:1623–8. 10.2967/jnumed.114.142109 25146124

[39] Norgren N Olsson M Nystrom H Ericzon BG de Tayrac M Genin E. Gene expression profile in hereditary transthyretin amyloidosis: Differences in targeted and source organs. Amyloid. (2014) 21:113. 10.3109/13506129.2014.894908 24601850 PMC4046871

[40] Qiang W Yau WM Lu JX Collinge J Tycko R. Structural variation in amyloid-beta fibrils from Alzheimer's disease clinical subtypes. Nature. (2017) 541:217–21. 10.1038/nature20814 28052060 PMC5233555

[41] Yamashita T Ando Y Ueda M Nakamura M Okamoto S Zeledon ME. Effect of liver transplantation on transthyretin Tyr114Cys-related cerebral amyloid angiopathy. Neurology. (2008) 70:123–8. 10.1212/01.wnl.0000287089.28847.b5 18180441

[42] Yamada M Tsukagoshi H Otomo E Hayakawa M. Cerebral amyloid angiopathy in the aged. J Neurol. (1987) 234:371–6. 10.1007/BF00314080 3655840

[43] Pasi M Pongpitakmetha T Charidimou A Singh SD Tsai HH Xiong L. Cerebellar microbleed distribution patterns and cerebral amyloid angiopathy. Stroke. (2019) 50:1727–33. 10.1161/STROKEAHA.119.024843 31159702 PMC6599711

[44] Charidimou A Law R Werring DJ. Amyloid “spells” trouble. Lancet. (2012) 380:1620. 10.1016/S0140-6736(12)61333-6 23122250

[45] Salvi F Pastorelli F Plasmati R Morelli C Rapezzi C Bianchi A. Brain microbleeds 12 years after orthotopic liver transplantation in Val30Met amyloidosis. J Stroke Cerebrovasc Dis. (2015) 24:e149–51. 10.1016/j.jstrokecerebrovasdis.2015.02.015 25802113

[46] Yamada Y Fukushima T Kodama S Shimizu H Kakita A Makino K. A case of cerebral amyloid angiopathy-type hereditary ATTR amyloidosis with Y69H (p. Y89H) variant displaying transient focal neurological episodes as the main symptom. Amyloid. (2019) 26:251–2. 10.1080/13506129.2019.1632829 31257920

[47] Mitrani LR De Los Santos J Driggin E Kogan R Helmke S Goldsmith J. Anticoagulation with warfarin compared to novel oral anticoagulants for atrial fibrillation in adults with transthyretin cardiac amyloidosis: comparison of thromboembolic events and major bleeding. Amyloid. (2021) 28:30–4. 10.1080/13506129.2020.1810010 32814468 PMC8018530

[48] Mockelind S Axelsson J Pilebro B Lindqvist P Suhr OB Sundstrom T. Quantification of cardiac amyloid with [F]Flutemetamol in patients with V30M hereditary transthyretin amyloidosis. Amyloid. (2020) 27:191–9. 10.1080/13506129.2020.1760237 32400202

